# Goal-directed fluid therapy on the postoperative complications of laparoscopic hepatobiliary or pancreatic surgery: An interventional comparative study

**DOI:** 10.1371/journal.pone.0315205

**Published:** 2024-12-18

**Authors:** Bon-Wook Koo, Ah-Young Oh, Hyo-Seok Na, Jiwon Han, Hyeong geun Kim

**Affiliations:** 1 Department of Anesthesiology and Pain Medicine, Seoul National University Bundang Hospital, Seongnam, South Korea; 2 Department of Anesthesiology and Pain Medicine, Seoul National University College of Medicine, Seoul, South Korea; IRCCS: IRCCS Ospedale San Raffaele, ITALY

## Abstract

**Background:**

Intraoperative fluid balance significantly affects patients’ outcomes. Goal-directed fluid therapy (GDFT) has reduced the incidence of major postoperative complications by 20% for 30 days after open abdominal surgery. Little is known about GDFT during laparoscopic surgery.

**Aim:**

We investigated whether GDFT affects the postoperative outcomes in laparoscopic hepatobiliary or pancreatic surgery compared with conventional fluid management.

**Methods:**

This interventional comparative study with a historical control group was performed in the tertiary care center. Patients were allocated to one of two groups. The GDFT (n = 147) was recruited prospectively and the conventional group (n = 228) retrospectively. In the GDFT group, fluid management was guided by the stroke volume (SV) and cardiac index (CI), whereas it had been performed based on vital signs in the conventional group. Propensity score (PS) matching was performed to reduce selection bias (n = 147 in each group). Postoperative complications were evaluated as primary outcome measures.

**Results:**

The amount of crystalloid used during surgery was less in the GDFT group than in the conventional group (5.1 ± 1.1 vs 6.3 ± 1.8 ml/kg/h, respectively; P <0.001), whereas the amount of colloid was comparable between the two groups. The overall proportion of patients who experienced any adverse events was 57.8% in the GDFT group and 70.1% in the conventional group (P = 0.038), of which the occurrence of pleural effusion was significantly lower in the GDFT group than in the conventional group (9.5% vs. 19.7%; P = 0.024). During the postoperative period, the proportion of patients admitted to the intensive care unit (ICU) was lower in the GDFT group than that in the conventional group after PS matching (4.1% vs 10.2%; P = 0.049).

**Conclusions:**

GDFT based on SV and CI resulted in a lower net fluid balance than conventional fluid therapy. The overall complication rate in laparoscopic hepatobiliary or pancreatic surgery decreased after GDFT, and the frequency of pleural effusion was the most affected.

## Introduction

Perioperative fluid management aims to maintain optimal intravascular volume, cardiac function, and tissue oxygen delivery [1, 2]. Intraoperative hypovolemia or hypervolemia is known to have a significant effect on patient recovery and prognosis [3, 4]. However, intraoperative fluid requirement depends on the patient’s condition, type of surgery, and surgical time; thus, standardization is difficult. Several methods, ranging from non-invasive to minimally invasive techniques as well as those necessitating advanced cardiac output monitoring and arterial cannulation, can be utilized to predict fluid responsiveness in patients, regardless of whether they are breathing spontaneously [5] or are on mechanical ventilation [6, 7].

Goal-directed fluid therapy (GDFT) determines the amount of fluid to be administered by continuously measuring indicators directly related to cardiac output and oxygen delivery, enabling more adequate fluid management than conventional methods relying on blood pressure, heart rate, or urine output. Preliminary studies typically apply protocols that assess the response to fluid infusion by observing variations in stroke volume (SV). Nevertheless, it is important to note that these protocols may exhibit variability contingent upon the specific subjects and research settings [1, 8–10]. FloTrac with EV1000^®^ platform (Edwards Lifesciences Corporation, Irvine, CA, USA) has the advantage of being used for continuous measurements of several cardiac parameters without additional invasive procedures when monitoring invasive arterial pressure. This minimally invasive cardiac monitoring system based on the arterial pressure waveform analysis has been clinically applied and validated in various conditions [11].

The progress in contemporary medical science has led to a paradigm shift in surgical methodologies, favoring a trend towards minimally invasive procedures. Consequently, laparoscopic or exoscopic surgeries are preferred over conventional open surgeries, owing to their diminished invasiveness [12, 13]. Numerous studies on GDFT have been conducted in major open abdominal surgeries like colorectal, vascular, liver [14–16] with various non-cardiac major surgeries during general anesthesia, and it has been reported that several postoperative complications, mortality, and hospital stay were decreased [17]. The use of cardiac output-guided hemodynamic therapy has not been shown to reduce the composite outcome of complications and 30-day mortality [18]. Whilst laparoscopic surgery significantly reduces surgical trauma compared to open surgery [19, 20], the elevated intra-abdominal pressure resulting from peritoneal insufflation can induce hemodynamic instability, leading to unfavorable neuroendocrine responses and outcomes. In major liver surgery the practice of maintaining low central venous pressure (CVP) during the transection phase aims to minimize bleeding. However, this approach, combined with increased intra-abdominal pressure, raises the risk of carbon dioxide gas embolism in laparoscopic surgery. Therefore, using stroke volume variation (SVV) instead of CVP as a measure of vascular bed filling is helpful for guiding fluid therapy during the transection phase [21]. In this particular scenario, the implementation of goal-directed fluid therapy (GDFT) has yielded favorable outcomes in fluid resuscitation [22].

Hepatobiliary and pancreatic surgeries procedures, including pancreatic duodenal resection or hepatic resection, are selectively performed across medical institutions, as they are recognized as complexity and significant inherent risk, with an average of 1400 hepato-biliary surgeries per year in high volume center [23]. The subsequent patient outcome assessment of these procedures requires a careful approach that encompasses the entire treatment continuum and considers both intraoperative fluid administration and hemodynamic status. However, the amount of fluid administered varies depending on the fluid restriction phase until parenchymal resection and the resuscitation phase after resection is complete for patients undergoing hepatectomy. Additionally, methods to reduce blood loss by maintaining a lower central venous pressure are being used, and the ERAS Society recommends euvolemia. Nevertheless, it is important to note that these protocols may exhibit variability contingent upon the specific subjects and research settings [16, 24]. Despite these efforts, little is known about GDFT during laparoscopic surgery with few studies done in hepatobiliary pancreatic surgeries [22]. Therefore, in this study, we investigated whether GDFT compared with conventional fluid therapy reduces postoperative complications, ICU admission and 90 days mortality in patients undergoing laparoscopic hepatobiliary or pancreatic surgery.

## Materials and methods

### Compliance with ethical guidelines

This interventional comparative study with a historical control group was approved in March 2016 by the Institutional Review Board (B-1604/343-002) and was registered with clinicaltrials.gov (NCT03169998). This study includes two groups, the GDFT and conventional groups. The GDFT group non-randomly included adult patients (>18 years) who were scheduled to undergo laparoscopic hepatobiliary or pancreatic surgery for more than 2 h. Hepatic surgery includes tumorectomy, minor segmentectomy as the resection of one or two liver segments and major segmentectomy as the resection of three or more liver segments, and lobectomy, while pancreatic surgery includes pylorus preserving pacreticoduodenectomy, pancreatectomy (central, distal, total). Bile duct surgery includes extended cholecystectomy and bile duct resection & anastomosis. Additionally, surgeries for both malignant and benign tumors are included. Written informed consent was obtained from all patients in the GDFT group. The GDFT group was recruited and acquired data prospectively between 9^th^ January 2017 and 7^th^ April 2020. Adult patients (>18 years old) who had undergone laparoscopic hepatobiliary or pancreatic surgery between 10^th^ January 2012 and 27^th^ December 2016 were selected retrospectively and assigned to the conventional group, and the data of conventional group were accessed through electric medical record for this research between 7^th^ April 2020 and 10^th^ July 2020. The written informed consent was exempted in the conventional group. We excluded of patients with end-stage renal disease, sepsis, pulmonary edema, congestive heart failure, arrhythmia, severe coagulopathy, and electrolyte imbalance that requires correction; Hyponatremia is defined as a serum sodium concentration of ≤135 mEq/L, and hypernatremia as ≥145 mEq/L, while hypokalemia is defined as a serum potassium concentration of ≤3.0 mEq/L, and hyperkalemia as ≥5.5 mEq/L. Moreover, when the surgical method was changed from laparoscopic to laparotomy, the patient was excluded.

Patients in the GDFT group received midazolam 0.03 mg/kg in the preoperative holding area. Anesthesia was induced with propofol 1–2 mg/kg, remifentanil target-controlled infusion starting at 3 ng/mL, and rocuronium 0.6 mg/kg IV. Anesthesia was maintained with desflurane, target-controlled infusion of remifentanil. Intraoperative anesthetic depth was monitored using the bispectral index (A-2000 BIS™ monitor; Aspect Medical Systems, Inc., Natick, MA, USA), which was maintained between 40–60. During general anesthesia, the ventilator settings were adjusted to maintain PaCO_2_ at 35–40 mmHg.

### GDFT management

**Fig 1:**
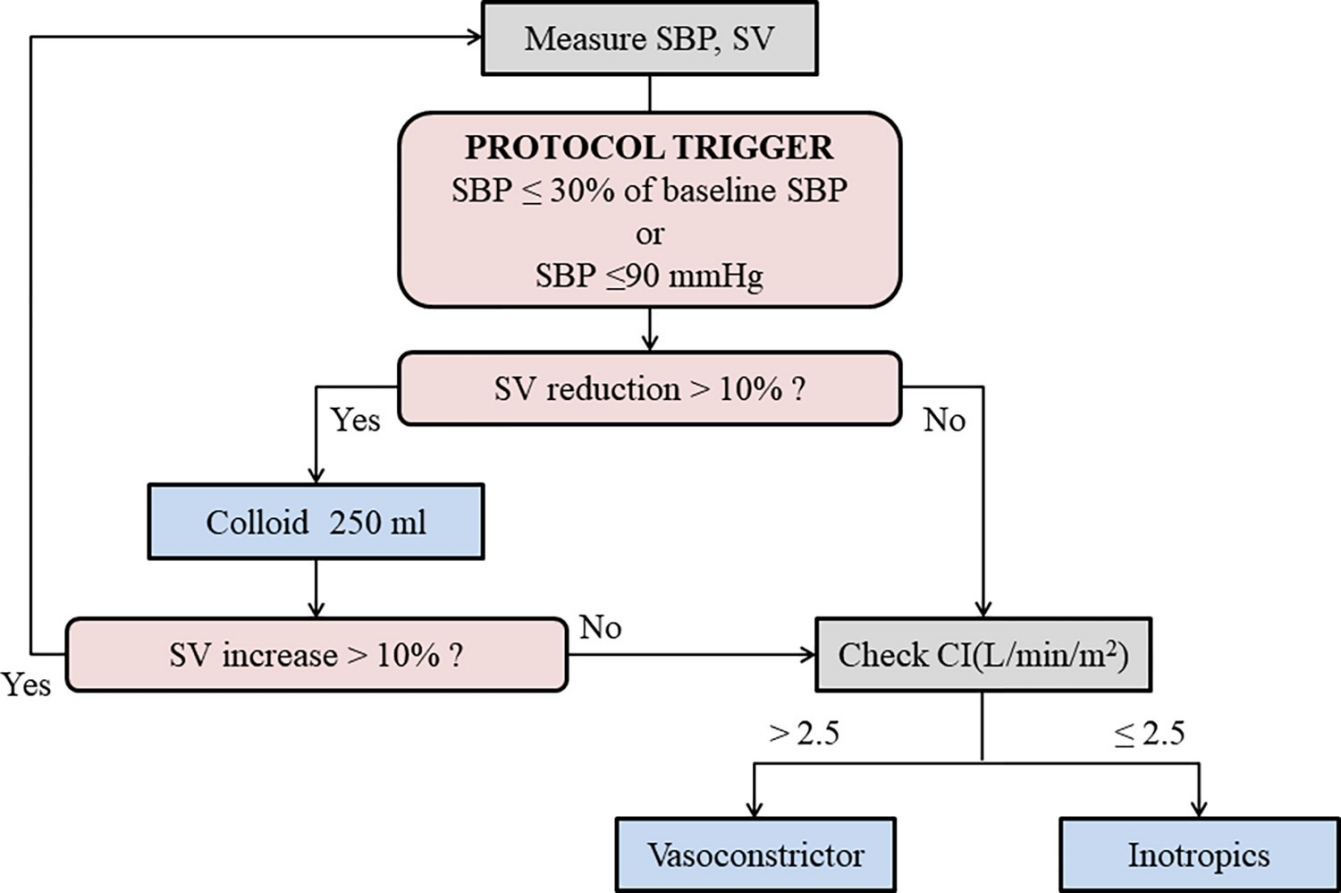
Flow diagram of intraoperative fluid management in goal-directed fluid therapy group.

A FloTrac sensor was connected to the cannulated arterial catheter. In addition to continuous arterial pressure, stroke volume (SV), cardiac index (CI), and stroke volume variation (SVV) were continuously monitored on the EV1000 platform (Edwards Lifescience Corp., Irvine, CA, USA) using a FloTrac sensor. An intravenous crystalloid was administered until the SVV reached the proper SVV zone. Thereafter, the target hemodynamic status during surgery was maintained at heart rate of 60-100/min and systolic arterial pressure within ± 30% of the preoperative baseline values. Crystalloid was administered to the patients at 4–5 ml/kg/h during the operation. The GDFT protocol is illustrated in Fig 1. If the systolic arterial pressure decreased by more than 30% of the baseline value or ≤ 90 mmHg, fluid and drug management were performed according to the SV and CI of EV1000 platform, which were validated to predict fluid responsiveness and to be acceptable for CI monitoring, respectively [25, 26]. When the SV was reduced by 10% or more compared to the prior value, 250 ml of colloid was administered. Hydroxyethyl starch 6% 130/0.4 in a balanced electrolyte solution was used as the colloid bolus, and the maximum infusion volume should not exceed 1500 ml. Thereafter, only crystalloid was used for volume replacement. When colloid loading was unable to restore the SV value or when hypotension occurred without SV reduction, vasoconstrictors or inotropic agents were administered according to the CI. If the CI value was 2.5 or higher, vasoconstrictor was administered, and if the CI value was 2.5 or lower, inotropics were administered. Phenylephrine was primarily chosen as a vasoconstrictor administered by bolus or continuous infusion. Ephedrine was first used as an inotropic agent, and norepinephrine was infused after the administration of ephedrine up to 15 mg.

### Transfusion

Red blood cells (RBCs) were transfused when hemoglobin levels were <8 g/dl. However, RBC transfusion was initiated despite Hb ≥8 g/dl in patients with coronary artery disease presenting with intraoperative electrocardiogram changes or continuous hypotension and tachycardia, hypoxemia, oligouria even with appropriate fluid volume. In addition, fresh frozen plasma was transfused when RBCs were transfused with more than 5 units for intraoperative bleeding or a hypocoagulable profile with prothrombin time of international normalized ratio >2.0 or greater than 1.5 times normal; an activated partial thromboplastin time of >60 s or greater than 2 times normal. Platelets were administered when RBCs had been transfused with more than 10 units for intraoperative bleeding or when platelet count was ≤50,000/mm^3^. Blood loss was estimated using a gauze visual analog (50% saturation: 80 ml, 100% saturation: 160 ml by 45 × 45 cm), and the amount of bleeding in the suction bottle was checked and measured periodically.

### Outcome measurement

Overall postoperative complications, such as acute kidney injury, stroke, delirium, pneumonia, atelectasis, deep vein thrombosis, sepsis, ileus, wound problems, myocardial infarction, bleeding, and urinary tract infection were evaluated as the primary outcomes until the discharge period. If the hospitalization period exceeded 90 days after surgery, complications were investigated only up to such time. Postoperative complications were defined according to the criteria of the European Perioperative Clinical Outcome definitions [27], in addition to the medical records and consultation with the relevant medical department. Secondary outcomes included the infused crystalloid or colloid volume during surgery, intraoperative urine output and blood loss, use of vasopressors and inotropics, intra- and postoperative transfusion, length of stay in the hospital or ICU, ICU admission and duration, readmission within 30 days postoperatively, reoperation within 90 days postoperatively, and death within 90 days postoperatively.

### Statistical analysis

Data were presented as mean ± standard deviation (SD) or number (%) with effect size (d), odds ratio (OR), or 95% confidence interval (95% CI), as appropriate. In a previous study showed difference of 15%, the rate of postoperative complications decreased from 39.8% to 24.8% when GDFT was performed on patients undergone open surgery [28]. In our center, the overall complication rate in laparoscopic hepatobiliary or pancreatic surgery was approximately 31%. Our hypothesis was the overall complication rate would be decreased by GDFT compared to conventional fluid therapy by 15%. To determine a difference of 15% in the incidence of postoperative complications with a statistical power of 80% and a type 1 error of 5%, we estimated that 145 patients were required in each group. Assuming an overall dropout rate of 10%, at least 162 patients per group were required.

The Kolmogorov–Smirnov test was used to test for normal distribution. Continuous data were tested using Student’s t-test. Binary data were tested using the chi-squared or Fisher’s exact test. Propensity score (PS) matching was performed to reduce the selection bias caused by differences in baseline characteristics between the two groups [29]. We used PS matching methods to balance the covariates between the two groups, with age, sex, height, weight, body mass index, American Society of Anesthesiology physical status, and organ of operation included in the PS model. After estimating the PS, patients were matched 1:1 without replacement using a nearest-neighbor approach. The balance in baseline characteristics and covariates after matching was evaluated using standardized mean differences. The paired t-test or McNemar’s test was performed according to continuous or binary data after propensity score matching. All statistical analyses were performed using SPSS with statistical significance was set at P <0.05.

## Results

**Fig 2:**
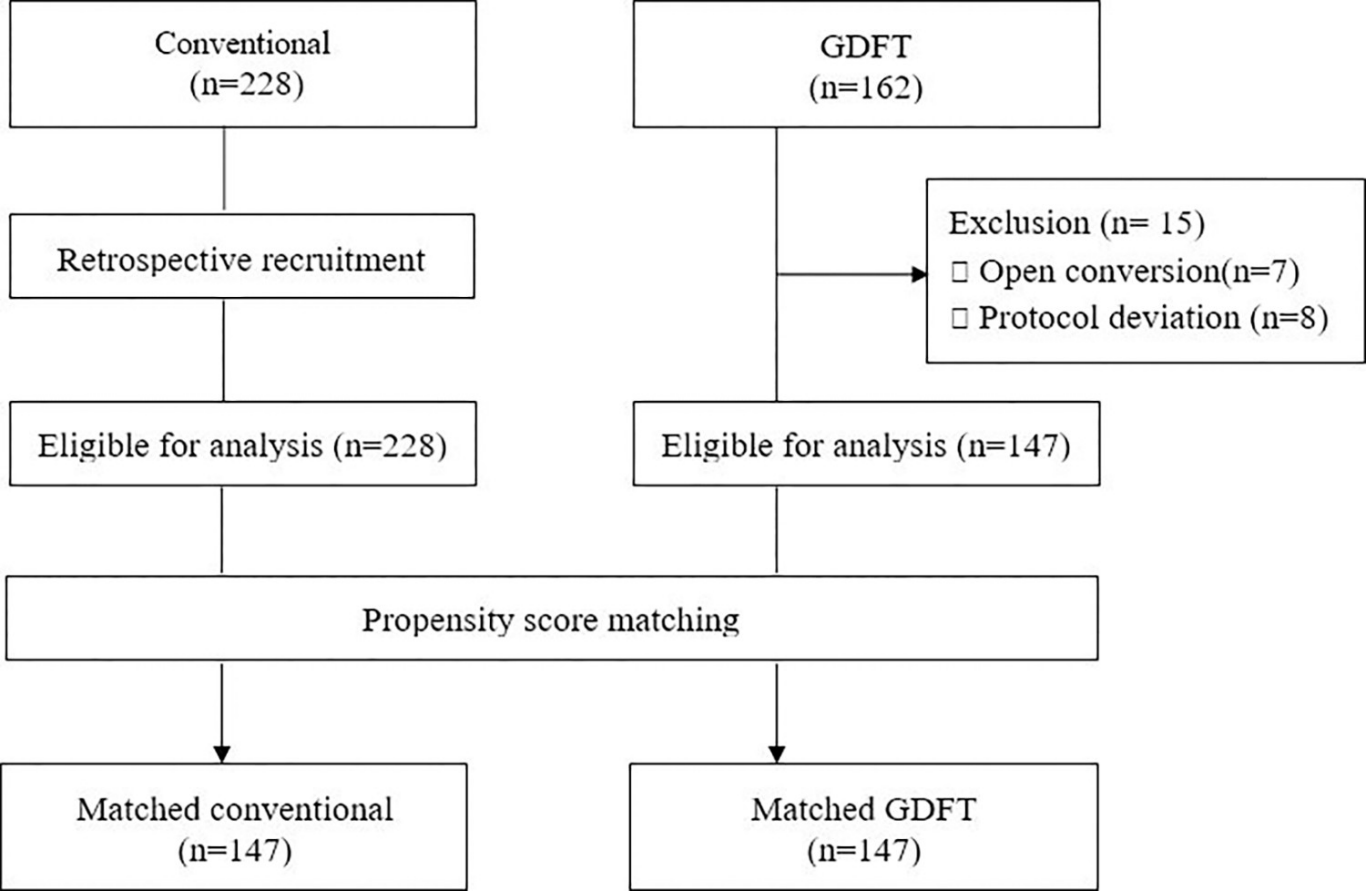
CONSORT diagram.

This study was conducted between 9^th^ January 2017 and 7^th^ April 2020 for GDFT group. A total of 162 and 228 patients were enrolled in the GDFT and conventional groups, respectively, while 15 patients were excluded from the GDFT group. This study conduced after PS matching, 147 patients were included in each group (Fig 2).

The characteristics of the patients, surgeries, and anesthesia are presented in Table 1. There were no statistically significant differences, except for the type of surgery before PS matching. The GDFT group accounted for a greater distribution of pancreatic surgeries than did the conventional group. The difference in surgery type was not observed after PS matching.

**Table 1:** The characteristic of patients, surgery, and anesthesia.

	Before propensity score matching				After propensity score matching			
	GDFT (n = 147)	Conventional (n = 228)	*P* value	SMD	GDFT (n = 147)	Conventional (n = 147)	*P* value	SMD
***Demographic data***								
Sex (M/F)	77/70 (52.4/47.6)	118/110 (51.8/48.2)	0.906	0.014	77/70 (52.4/47.6)	80/67 (54.4/45.6)	0.798	0.045
Age (years)	61.5 ± 11.82	58.9 ± 14.9	0.063	0.189	61.5 ± 11.82	62.3 ± 13.6	0.573	0.0636
Weight (kg)	65.0 ± 11.9	63.1 ± 11.2	0.125	0.166	65.0 ± 11.9	63.8 ± 11.4	0.374	0.103
Height (cm)	162.0 ± 9.5	162.0 ± 8.9	0.942	0	162.0 ± 9.5	161.8 ± 9.2	0.901	0.021
BMI (kg/m^2^)	24.7 ± 3.3	24.0 ± 3.5	0.059	0.205	24.7 ± 3.3	24.3 ± 3.4	0.292	0.120
ASA (I/II/III)	36/98/13 (24.5/66.7/8.8)	69/148/11 (30.3/64.9/4.8)	0.186	0.180	36/98/13 (24.5/66.7/8.8)	29/108/10 (19.7/73.5/6.8)	0.619	0.052
***Surgical data***								
Type of operation Pancreas/Liver/Biliary tract	77/59/11 (52.4/40.1/7.5)	86/95/47 (37.7/41.7/20.6)	0.001	0.396	77/59/11 (52.4/40.1/7.5)	75/62/10 (51.0/42.2/6.8)	0.815	0.011
Operation time (min)	278.0 ± 123.2	300.1 ± 136.0	0.112	0.169	278.0 ± 123.2	307.8 ± 146.1	0.077	0.221
***Anesthetic data***								
Type of anesthesia Inhalation / TIVA	140/7 (95.2/4.8)	213/15 (93.4/6.6)	0.465	0.189	140/7 (95.2/4.8)	140/7 (95.2/4.8)	1.000	0
Anesthesia time (min)	323.8 ± 128.6	348.5 ± 137.6	0.082	0.184	323.8 ± 128.6	355.8 ± 147.7	0.063	0.231

Values represent mean ± standard deviation or number (%).

GDFT, goal-directed fluid therapy; BMI, body mass index; ASA, physical status of American Society of Anesthesiologists; TIVA, total intravenous anesthesia; SMD, standardized mean difference.

Table 2 summarizes the results of fluid therapy, drug medication, and transfusion before and after PS-matching. In the PS matching groups, the amount of crystalloid used during surgery was less in the GDFT group (5.1 ± 1.1 ml/kg/h) than in the conventional group (6.3 ± 1.8 ml/kg/h) (*d* = 0.56; 95% CI = -1.56 and -0.86; P <0.001), whereas the amount of colloid was comparable between the two groups. Urine output was significantly lower in the GDFT group (0.7 ± 0.4 ml/kg/h) than in the conventional group (0.7 ± 0.4 vs 0.9 ± 0.7 ml/kg/h, respectively; P = 0.005) (d = 0.23; 95% CI = -0.34 and -0.06; P = 0.005). Ephedrine was used less frequently (56 [38.1%] vs. 93 [63.3%] for the GDFT vs. the conventional group; OR = 0.35; 95% CI = 0.20 and 0.59; P <0.001), while phenylephrine was used more frequently (111 [75.5%] vs 75 [51.0%] for the GDFT vs. the conventional group; OR = 2.90; 95% CI = 1.69 and 5.17; P <0.001) in the GDFT group than in the conventional group. The incidence of norepinephrine use was higher in the GDFT group before PS matching; however, it became comparable after PS matching. Transfusion profiles were comparable between the two groups before and after PS matching.

**Table 2:** Intraoperative fluid management, drug medication, and transfusion profiles.

	Before propensity score matching				After propensity score matching			
	GDFT (n = 147)	Conventional (n = 228)	*P* value	SMD	GDFT (n = 147)	Conventional (n = 147)	*P* value	SMD
Crystalloid (ml/kg/h)	5.1 ± 1.1	6.3 ± 1.8	<0.001^a^	0.767	5.1 ± 1.1	6.3 ± 1.8	<0.001^a^	0.805
Colloid (ml/kg/h)	0.9 ± 1.2	1.0 ± 1.2	0.523^a^	0.083	0.9 ± 1.2	1.0 ± 1.1	0.589^a^	0.087
Estimate blood loss (ml)	349.9 ± 494.5	422.2 ± 476.1	0.158^a^	0.150	349.9 ± 494.5	434.1 ± 488.3	0.160^a^	0.171
Urine (ml/kg/h)	0.7 ± 0.4	0.8 ± 0.7	0.001^a^	0.167	0.7 ± 0.4	0.9 ± 0.7	0.005^a^	0.351
Ephedrine	56 (38.1)	139 (61.0)	<0.001^b^	0.514	56 (38.1)	93 (63.3)	<0.001^b^	0.567
Phenylephrine	111 (75.5%)	109 (47.8)89	<0.001^b^	0.674	111 (75.5)	75 (51.0)	<0.001^b^	0.598
Norepinephrine	30 (20.4)	21 (9.2)	0.002^b^	0.511	30 (20.4)	18 (12.2)	0.104^b^	0.336
Intraop. RBC	8 (5.4)	21 (9.2)	0.182^a^	0.313	8 (5.4)	15 (10.2)	0.210^a^	0.375
Intraop. RBC (unit)†	3.0 ± 1.3†	2.1 ± 1.2†	0.074^a^	0.726	1.0 ± 1.6†	1.4 ± 1.4†	0.536^a^	0.266
Postop. RBC	7 (4.8)	12 (5.3)	0.829^a^	0.058	7 (4.8)	9 (6.1)	0.804^a^	0.173
Postop. RBC (unit)†	2.9 ± 2.4†	3.5 ± 4.1†	0.710^a^	0.170	1.3 ± 2.1†	1.2 ± 1.2†	0.935^a^	0.059
Intraop. FFP	2 (1.4)	3 (1.3)	1.000^a^	0.019	2 (1.4)	3 (2.0)	1.000^a^	0.227
Postop. FFP	3 (2.0)	2 (0.9)	0.384^a^	0.472	3 (2.0)	0 (0)	NA^a^	NA
Intraop. Platelets	1 (0.7)	0 (0)	0.392^a^	NA	1 (0.7)	0 (0)	NA^a^	NA
Postop. Platelets	0 (0)	1 (0.4)	1.000^a^	NA	0 (0)	0 (0)	NA^a^	NA

Values represent mean ± standard deviation or number (%).

GDFT, goal-directed fluid therapy; RBC, red blood cell; FFP, fresh frozen plasma; Intraop, intraoperative; postop, postoperative; NA, not applicable; SMD, standardized mean difference

†Mean ± standard deviation was obtained only for patients who received RBC transfusions.

*p* values were calculated using

^a^ student t-test

^b^ chi-squared or Fisher’s exact test.

The proportion of patients who experienced any adverse events was 85 (57.8%) in the GDFT group and 103 (70.1%) in the conventional group after PS matching (OR 0.58; 95% CI = 0.34 and 0.97; P = 0.038) (Table 3). When evaluating the incidence of each complication, the occurrence of pleural effusion was significantly lower in the GDFT group (n = 14 [9.5%]) than that in the conventional group (n = 29, 19.7%) (OR 0.44; 95% CI = 0.21 and 0.91; P = 0.024). AKI more frequently occurred in the GDFT group than in the conventional group before PS matching; however, after PS matching, the incidence of AKI was not statistically significant (n = 14 [9.5%] for the CDFT vs. n = 6 [4.1%] for the conventional group) (OR 2.6; 95% CI = 0.87 and 9.32; P = 0.096). There were no significant differences observed in other complications between the two groups.

**Table 3:** Postoperative complications.

	Before propensity score matching				After propensity score matching			
	GDFT (n = 147)	Conventional (n = 228)	*P* value^a^	SMD	GDFT (n = 147)	Conventional (n = 147)	*P* value^a^	SMD
Overall	68 (46.3)	126 (55.3)	0.088	0.199	68 (46.3)	103 (70.1)	0.038	0.295
AKI	14 (9.5)	10 (4.4)	0.047	0.458	14 (9.5)	6 (4.1)	0.096	0.499
Stroke	1 (0.7)	1 (0.4)	1.000	0.243	1 (0.7)	1 (0.7)	1.000	0
Delirium	1 (0.7)	0 (0)	0.392	NA	1 (0.7)	0 (0)	NA	NA
Atelectasis	19(12.9)	33 (14.5)	0.672	0.072	19 (12.9)	21 (14.3)	0.864	0.064
Pleural effusion	14 (9.5)	41 (18.0)	0.025	0.405	14 (9.5)	29 (19.7)	0.024	0.468
Pneumonia	1 (0.7)	3 (1.3)	1.000	0.367	1 (0.7)	2(1.4)	1.000	0.386
DVT	4 (2.7)	12 (5.3)	0.234	0.378	4 (2.7)	6 (4.1)	0.754	0.231
Sepsis	0 (0)	5 (2.2)	0.161	NA	0 (0)	2 (1.4)	NA	NA
Ileus	14 (9.5)	28 (12.3)	0.409	0.157	14 (9.5)	13 (8.8)	1.000	0.045
Wound complication	19 (12.9)	46 (20.2)	0.070	0.293	19 (12.9)	30 (20.4)	0.126	0.301
MI	0 (0)	0 (0)	NA	NA	0 (0)	0 (0)	NA	NA
Postoperative bleeding	5 (3.4)	3 (1.3)	0.272	0.535	5 (3.4)	1 (0.7)	0.125	0.903
UTI	1 (0.7)	2 (0.9)	1.000	0.141	1 (0.7)	2 (1.4)	1.000	0.386

Values represent number (%).

GDFT, goal-directed fluid therapy; AKI, acute kidney injury; DVT, deep vein thrombosis; MI, myocardial infarction; UTI, urinary tract infection; SMD, standardized mean difference; NA, not applicable

^a^*p* values were calculated using the chi-squared or Fisher’s exact test.

During the postoperative period, the proportion of patients admitted to the ICU was lower in the GDFT group (n = 6 [4.1%]) than in the conventional group after PS matching (n = 15 [10.2%]) (OR 0.31; 95% CI = 0.07 and 0.99; P = 0.049) (Table 4). Other postoperative outcome variables, such as readmission, reoperation, and death, were comparable between the two groups.

**Table 4:** Postoperative outcomes.

	Before propensity score matching				After propensity score matching			
	GDFT (n = 147)	Conventional (n = 228)	*P* value	SMD	GDFT (n = 147)	Conventional (n = 147)	*P* value	SMD
ICU admission	6 (4.1)	20 (8.8)	0.081^b^	0.446	6 (4.1)	15 (10.2)	0.049^b^	0.542
ICU stay (day)†	3.2 ± 3.6†	3.6 ± 5.4†	0.867^a^	0.079	1.0 ± 1.7†	1.9 ± 1.6†	0.187^a^	0.545
Readmission within 30 days	15 (10.2)	22 (9.6)	0.860^b^	0.034	15 (10.2)	15 (10.2)	1.000^b^	0
Reoperation within 90 days	0	4 (1.8)	0.303^b^	NA	0 (0)	2(1.4)	NA^b^	NA
LOS in hospital (day)	9.3 ± 6.1	10.3 ± 9.2	0.249^a^	0.123	9.3 ± 6.1	10.1 ± 5.7	0.953^a^	0.136
Death within 90 days	1 (0.7)	2 (0.9)	1.000^b^	0.141	1 (0.7)	1 (0.7)	1.000^b^	0

Values represent mean ± standard deviation or number (%).

GDFT, goal-directed fluid therapy; ICU, intensive care unit; LOS, length of hospital stay; SMD, standardized mean difference

†Mean ± standard deviation was obtained only for patients who admitted at ICU.

*p* values were calculated using

^a^ student t-test

^b^ chi-squared or Fisher’s exact test.

## Discussion

In this study, we confirmed that GDFT reduced the overall incidence of postoperative complications, particularly pleural effusion and ICU admissions, in laparoscopic hepatobiliary and pancreatic surgeries.

Postoperative pulmonary complications, including pleural effusion are the second most common postoperative complications following wound infection [30, 31]. Although there are reports that laparoscopic surgery reduces postoperative pulmonary complications compared to open surgery [32, 33], a key finding of this study is that GDFT provided an additional benefit by reducing the incidence of pleural effusion in laparoscopic hepatobiliary and pancreatic surgeries. The reason for the GDFT decrease in pleural effusion was not evaluated in this study, but various causes of postoperative pleural effusion have been suggested, such as hypervolemia, pulmonary embolism, atelectasis, abdominal fluid, irritated diaphragm, subphrenic abscess, or pancreatitis [34]. In our study, despite similar colloid administration between the two groups, GDFT allowed for reduced crystalloid use and more frequent administration of inotropes or vasoconstrictors during surgery. This approach enabled rapid correction of hypoperfusion and ensured adequate oxygen delivery, preventing systemic inflammation that could increase capillary permeability. Consequently, GDFT may contribute to a reduction in pleural effusion incidence by preventing fluid overload and maintaining hemodynamic stability, compared to conventional fluid management. The results following PS matching further objectively support the association between GDFT and the reduction in postoperative pleural effusion.

Intraoperative fluid overload is a concern, but excessive fluid restriction can also lead to hypovolemia, increasing the risk of postoperative acute kidney injury (AKI). Although GDFT reduces intraoperative fluid requirements in laparoscopic surgery [35–37], it simultaneously monitors cardiovascular parameters to manage fluids and medications appropriately. However, the effects of GDFT on postoperative AKI remain controversial. Vaca et al. have reported that GDFT did not decrease the incidence of AKI after colorectal surgery [38], whereas it was reported to reduce AKI incidence in laparoscopic liver resection [39]. In the present study, the intraoperative urine output was lower in the GDFT group than in the conventional group. However, the incidence of AKI was comparable between the two groups after PS matching, although it had appeared to be different before PS matching. The most important confounder was the type of operation. Before PS matching, the GDFT group included more pancreatic surgeries than the conventional group did, which was controlled by the PS matching process. Thus, the AKI was comparable between the GDFT and the conventional group, respectively, after PS matching.

Interestingly, the ICU admission rate was lower in the GDFT group. The leading cause of ICU admission was a compromised cardiovascular condition, followed by respiratory problems, bleeding, and severe subcutaneous emphysema. Although only a few studies have investigated ICU admission after GDFT, there are reports that the use of GDFT in open abdominal surgery reduces the incidence of postoperative pneumonia and wound infection and significantly shortens ICU stay [28, 40]. However, there are also reports that GDFT does not have a significant impact on ICU admission [41]. In our study, the criteria for ICU admission were not specifically established; instead, the decision was made according to routine clinical criteria. Although it cannot be concluded that GDFT reduces ICU admission, the impact of GDFT on ICU admission should not be completely excluded.

Following lung complications, the second and the third common complications were infectious complication and ileus. Postoperative infections, encompassing surgical site infections, in patients with hepatocellular carcinoma contribute to complications that are associated with an elevated long-term risk of cancer recurrence and mortality [42]. GDFT was reported to be associated with a reduction in surgical site infection after abdominal surgery [43], and improved tissue perfusion and oxygenation have been suggested as possible mechanisms [44, 45]. Intravenous fluid overload during surgery may exert deleterious effects on gastrointestinal function, which can cause ileus, gastrointestinal edema, or local infection after surgery [3, 46, 47]. However, our results did not show significant differences in wound complications or ileus events. In addition, there were few other complications that did not differ between the two groups. Although the evidence is insufficient, laparoscopic hepatobiliary or pancreatic surgery has been found to exhibit similar or better postoperative outcomes than those of open surgery [48–50].

GDFT has been associated with better recovery and fewer postoperative complications, not only following open abdominal and major non-cardiac surgeries [10, 51, 52], but also in laparoscopic surgeries [35–37]. However, few clinical studies investigating the effects of GDFT in laparoscopic hepatobiliary and pancreatic surgeries [39]. This can be attributed to the high complexity and invasiveness of these procedures, the frequent need for laparotomy, significant intraoperative blood loss, and the elevated risk of postoperative complications. In this study, unlike in upper and lower gastrointestinal surgeries [22], the frequency of vasopressor use during surgery was higher in GDFT patients. This discrepancy is thought to result from the inherent hemodynamic instability and longer operative times characteristic of hepatobiliary and pancreatic surgeries, which may have also contributed to the lack of reduction in hospital stay. If hypotension could be predicted, preventive measures could be implemented in advance, thereby mitigating the impact of intraoperative hypotension. A recent study using randomized trial demonstrated that applying an early warning system (EWS) developed through machine learning, in contrast to conventional care, significantly reduced the occurrence of low blood pressure during surgery [53]. Therefore, the necessity of GDFT becomes even more emphasized. However, there are another reports that the effect of GDFT is insignificant depending on the type of surgery or on medical disease [9, 36, 54, 55]. In septic patients presenting to the emergency department, the systematic and structured approach of early goal-directed therapy (EGDT) facilitated the timely recognition and management of higher severity of illness. This approach enabled prompt and aggressive resuscitation efforts, resulting in improved hemodynamic stability and a significant reduction in one-year mortality [56].

This study had several limitations. First, there was a possibility of bias in the conventional group because the data of the conventional group were collected retrospectively. However, the postoperative complications were evaluated using consistent criteria; thus, their incidence was not considered to be significantly biased. Second, the surgeons’ surgical techniques may have improved over time. The surgeons in charge of hepatobiliary and pancreatic surgery did not change during the study period, and all were experts who had been performing surgery in that field for more than 10 years. Therefore, it is expected that there will not be much difference in surgical skills over time. Third, most of the predominant enrollment of patients categorized as ASA I or II, with a comparatively minimal representation of those in the high-risk category exhibiting cardiovascular comorbidities. It is noteworthy that individuals with such medical profiles are predisposed to an augmented likelihood of admission to ICU, concomitant with an elevated susceptibility to postoperative complications and mortality [57]. Consequently, the discernible efficacy of Goal-Directed Fluid Therapy (GDFT) may have been more accentuated within this subset of patients. Fourth, practical challenges related to the implementation of the GDFT protocol, particularly regarding costs, have not been thoroughly investigated. There is a significant correlation between the occurrence of postoperative complications and the increase of costs in hepatobiliary and pancreatic surgeries [58–60]. If GDFT reduces postoperative complications for patients, it is crucial to consider the cost-effectiveness of medical device usage. To achieve this, health economic analyses such as a systematic review of cost and cost-effectiveness studies or the construction of a de novo cost-effectiveness model are required. Fifth, this study was conducted at a single institution, and external validation has not been performed to assess whether the findings can be generalized to alternative anesthesia protocols and other settings. However, the fact that clinical conditions were consistently controlled and that the criteria for judging postoperative complications were equally applied may have been advantageous in interpreting the results. This can be addressed by further studies involving multiple institutions [61]. Finally, there was no evaluation of microcirculatory indicators. Administration of fluid therapy based on hemodynamic indicators in goal-directed fluid therapy (GDFT) may lead to microcirculatory tissue oxygenation dysfunction, even when utilizing macro-circulatory indicators. Recently, monitoring methods evaluating the status of microcirculation, such as sublingual microscopy, vascular occlusion test, and laser doppler flowmetry, have been introduced [62]. These tests have the advantage of being able to examine the microcirculation specific to lesions in a relatively simple and non-invasive manner, different from GDFT. It is anticipated that future research will focus on the evaluation of these medical devices for GDFT.

## Conclusions

In conclusion, GDFT in laparoscopic hepatobiliary and pancreatic surgery appears to reduce postoperative complications, particularly pleural effusion, and ICU admissions. The findings support the use of GDFT as a strategy to optimize fluid management and hemodynamic stability during these complex procedures. Future research should focus on predictive fluid therapy models to prevent intraoperative hypotension and evaluate the broader applicability and cost-effectiveness of GDFT in diverse surgical and clinical settings.

## Supporting information

S1 Table Intraoperative fluid management, drug medication, and transfusion profiles before propensity score matching. (DOCX)

S2 Table Postoperative complications after propensity score matching before propensity score matching. (DOCX)

S3 Table Postoperative outcomes before propensity score matching. (DOCX)

S4 Table Intraoperative fluid management, drug medication, and transfusion profiles after propensity score matching. (DOCX)

S5 Table Postoperative complications after propensity score matching after propensity score matching. (DOCX)

S6 Table Postoperative outcomes after propensity score matching. (DOCX)

S1 File CONSORT checklist. (DOC)

S2 File IRB study protocol Korean version. (DOCX)

S3 File Study protocol English translation version. (DOCX)

S4 File IRB approval file. (PDF)

S5 File Raw data. (XLSX)
