# Supplementary material for: Goal-directed fluid therapy on the postoperative complications of laparoscopic hepatobiliary or pancreatic surgery: An interventional comparative study
Source: PLoS One. 2024 Dec 18;19(12):e0315205. doi: 10.1371/journal.pone.0315205 (PMC11654985; doi:10.1371/journal.pone.0315205)
Supplement: S1 Table — (DOCX) [file pone.0315205.s001.docx]

Table 1. Intraoperative fluid management, drug medication, and transfusion profiles before propensity score matching.

|  | GDFT  (n = 147) | Conventional  (n = 228) | *P* value | SMD |
| --- | --- | --- | --- | --- |
| Crystalloid (ml/kg/h) | 5.1 ± 1.1 | 6.3 ± 1.8 | <0.001^a^ | 0.767 |
| Colloid (ml/kg/h) | 0.9 ± 1.2 | 1.0 ± 1.2 | 0.523^a^ | 0.083 |
| Estimate blood loss (ml) | 349.9 ± 494.5 | 422.2 ± 476.1 | 0.158^a^ | 0.150 |
| Urine (ml/kg/h) | 0.7 ± 0.4 | 0.8 ± 0.7 | 0.001^a^ | 0.167 |
| Ephedrine | 56 (38.1) | 139 (61.0) | <0.001^b^ | 0.514 |
| Phenylephrine | 111 (75.5%) | 109 (47.8)89 | <0.001 ^b^ | 0.674 |
| Norepinephrine | 30 (20.4) | 21 (9.2) | 0.002 ^b^ | 0.511 |
| Intraop. RBC | 8 (5.4) | 21 (9.2) | 0.182 ^a^ | 0.313 |
| Intraop. RBC (unit)† | 3.0 ± 1.3† | 2.1 ± 1.2† | 0.074 ^a^ | 0.726 |
| Postop. RBC | 7 (4.8) | 12 (5.3) | 0.829 ^a^ | 0.058 |
| Postop. RBC (unit)† | 2.9 ± 2.4† | 3.5 ± 4.1† | 0.710 ^a^ | 0.170 |
| Intraop. FFP | 2 (1.4) | 3 (1.3) | 1.000 ^a^ | 0.019 |
| Postop. FFP | 3 (2.0) | 2 (0.9) | 0.384 ^a^ | 0.472 |
| Intraop. Platelets | 1 (0.7) | 0 (0) | 0.392 ^a^ | NA |
| Postop. Platelets | 0 (0) | 1 (0.4) | 1.000 ^a^ | NA |

Values represent mean ± standard deviation or number (%).

GDFT, goal-directed fluid therapy; RBC, red blood cell; FFP, fresh frozen plasma; Intraop, intraoperative; postop, postoperative; NA, not applicable; SMD, standardized mean difference

†Mean ± standard deviation was obtained only for patients who received RBC transfusions.

p values were calculated using ^a^ student t-test; ^b^ chi-squared or Fisher’s exact test
