# Supplementary material for: Goal-directed fluid therapy on the postoperative complications of laparoscopic hepatobiliary or pancreatic surgery: An interventional comparative study
Source: PLoS One. 2024 Dec 18;19(12):e0315205. doi: 10.1371/journal.pone.0315205 (PMC11654985; doi:10.1371/journal.pone.0315205)
Supplement: S2 Table — (DOCX) [file pone.0315205.s002.docx]

Table 2. Postoperative complications after propensity score matching before propensity score matching.

|  | GDFT  (n = 147) | Conventional  (n = 228) | *P* value^a^ | SMD |
| --- | --- | --- | --- | --- |
| Overall | 68 (46.3) | 126 (55.3) | 0.088 | 0.199 |
| AKI | 14 (9.5) | 10 (4.4) | 0.047 | 0.458 |
| Stroke | 1 (0.7) | 1 (0.4) | 1.000 | 0.243 |
| Delirium | 1 (0.7) | 0 (0) | 0.392 | NA |
| Atelectasis | 19(12.9) | 33 (14.5) | 0.672 | 0.072 |
| Pleural effusion | 14 (9.5) | 41 (18.0) | 0.025 | 0.405 |
| Pneumonia | 1 (0.7) | 3 (1.3) | 1.000 | 0.367 |
| DVT | 4 (2.7) | 12 (5.3) | 0.234 | 0.378 |
| Sepsis | 0 (0) | 5 (2.2) | 0.161 | NA |
| Ileus | 14 (9.5) | 28 (12.3) | 0.409 | 0.157 |
| Wound complication | 19 (12.9) | 46 (20.2) | 0.070 | 0.293 |
| MI | 0 (0) | 0 (0) | NA | NA |
| Postoperative bleeding | 5 (3.4) | 3 (1.3) | 0.272 | 0.535 |
| UTI | 1 (0.7) | 2 (0.9) | 1.000 | 0.141 |

Values represent mean ± standard deviation or number (%).

GDFT, goal-directed fluid therapy; AKI, acute kidney injury; DVT, deep vein thrombosis; MI, myocardial infarction; UTI, urinary tract infection; SMD, standardized mean difference; NA, not applicable.

^a^p values were calculated using the chi-squared or Fisher’s exact test.
