# Supplementary material for: Goal-directed fluid therapy on the postoperative complications of laparoscopic hepatobiliary or pancreatic surgery: An interventional comparative study
Source: PLoS One. 2024 Dec 18;19(12):e0315205. doi: 10.1371/journal.pone.0315205 (PMC11654985; doi:10.1371/journal.pone.0315205)
Supplement: S4 Table — (DOCX) [file pone.0315205.s004.docx]

Table 4. Intraoperative fluid management, drug medication, and transfusion profiles after propensity score matching.

|  | GDFT  (n = 147) | Conventional  (n = 147) | *P* value | SMD |
| --- | --- | --- | --- | --- |
| Crystalloid (ml/kg/h) | 5.1 ± 1.1 | 6.3 ± 1.8 | <0.001 ^a^ | 0.805 |
| Colloid (ml/kg/h) | 0.9 ± 1.2 | 1.0 ± 1.1 | 0.589 ^a^ | 0.087 |
| Estimate blood loss (ml) | 349.9 ± 494.5 | 434.1 ± 488.3 | 0.160 ^a^ | 0.171 |
| Urine (ml/kg/h) | 0.7 ± 0.4 | 0.9 ± 0.7 | 0.005 ^a^ | 0.351 |
| Ephedrine | 56 (38.1) | 93 (63.3) | <0.001 ^b^ | 0.567 |
| Phenylephrine | 111 (75.5) | 75 (51.0) | <0.001 ^b^ | 0.598 |
| Norepinephrine | 30 (20.4) | 18 (12.2) | 0.104 ^b^ | 0.336 |
| Intraop. RBC | 8 (5.4) | 15 (10.2) | 0.210 ^a^ | 0.375 |
| Intraop. RBC (unit)† | 1.0 ± 1.6† | 1.4 ± 1.4† | 0.536 ^a^ | 0.266 |
| Postop. RBC | 7 (4.8) | 9 (6.1) | 0.804 ^a^ | 0.173 |
| Postop. RBC (unit)† | 1.3 ± 2.1† | 1.2 ± 1.2† | 0.935 ^a^ | 0.059 |
| Intraop. FFP | 2 (1.4) | 3 (2.0) | 1.000 ^a^ | 0.227 |
| Postop. FFP | 3 (2.0) | 0 (0) | NA ^a^ | NA |
| Intraop. Platelets | 1 (0.7) | 0 (0) | NA ^a^ | NA |
| Postop. Platelets | 0 (0) | 0 (0) | NA ^a^ | NA |

Values represent mean ± standard deviation or number (%).

GDFT, goal-directed fluid therapy; RBC, red blood cell; FFP, fresh frozen plasma; Intraop, intraoperative; postop, postoperative; NA, not applicable; SMD, standardized mean difference

†Mean ± standard deviation was obtained only for patients who received RBC transfusions.

p values were calculated using ^a^ student t-test; ^b^ chi-squared or Fisher’s exact test
