# Supplementary material for: Goal-directed fluid therapy on the postoperative complications of laparoscopic hepatobiliary or pancreatic surgery: An interventional comparative study
Source: PLoS One. 2024 Dec 18;19(12):e0315205. doi: 10.1371/journal.pone.0315205 (PMC11654985; doi:10.1371/journal.pone.0315205)
Supplement: S5 Table — (DOCX) [file pone.0315205.s005.docx]

|  | GDFT  (n = 147) | Conventional  (n = 147) | *P* value^a^ | SMD |
| --- | --- | --- | --- | --- |
| Overall | 85 (57.8) | 103 (70.1) | 0.038 | 0.295 |
| AKI | 14 (9.5) | 6 (4.1) | 0.096 | 0.499 |
| Stroke | 1 (0.7) | 1 (0.7) | 1.000 | 0 |
| Delirium | 1 (0.7) | 0 (0) | NA | NA |
| Atelectasis | 19 (12.9) | 21 (14.3) | 0.864 | 0.064 |
| Pleural effusion | 14 (9.5) | 29 (19.7) | 0.024 | 0.468 |
| Pneumonia | 1 (0.7) | 2(1.4) | 1.000 | 0.386 |
| DVT | 4 (2.7) | 6 (4.1) | 0.754 | 0.231 |
| Sepsis | 0 (0) | 2 (1.4) | NA | NA |
| Ileus | 14 (9.5) | 13 (8.8) | 1.000 | 0.045 |
| Wound complication | 19 (12.9) | 30 (20.4) | 0.126 | 0.301 |
| MI | 0 (0) | 0 (0) | NA | NA |
| Postoperative bleeding | 5 (3.4) | 1 (0.7) | 0.125 | 0.903 |
| UTI | 1 (0.7) | 2 (1.4) | 1.000 | 0.386 |

Table 5. Postoperative complications after propensity score matching after propensity score matching.

Values represent mean ± standard deviation or number (%).

GDFT, goal-directed fluid therapy; AKI, acute kidney injury; DVT, deep vein thrombosis; MI, myocardial infarction; UTI, urinary tract infection; SMD, standardized mean difference; NA, not applicable

^a^p values were calculated using the chi-squared or Fisher’s exact test
