# Supplementary material for: Goal-directed fluid therapy on the postoperative complications of laparoscopic hepatobiliary or pancreatic surgery: An interventional comparative study
Source: PLoS One. 2024 Dec 18;19(12):e0315205. doi: 10.1371/journal.pone.0315205 (PMC11654985; doi:10.1371/journal.pone.0315205)
Supplement: S6 Table — (DOCX) [file pone.0315205.s006.docx]

Table 6. Postoperative outcomes after propensity score matching.

|  | GDFT  (n = 147) | Conventional  (n = 147) | *P* value | SMD |
| --- | --- | --- | --- | --- |
| ICU admission | 6 (4.1) | 15 (10.2) | 0.049 ^b^ | 0.542 |
| ICU stay (day)† | 1.0 ± 1.7† | 1.9 ± 1.6† | 0.187 ^a^ | 0.545 |
| Readmission within 30 days | 15 (10.2) | 15 (10.2) | 1.000 ^b^ | 0 |
| Reoperation within 90 days | 0 (0) | 2(1.4) | NA ^b^ | NA |
| LOS in hospital (day) | 9.3 ± 6.1 | 10.1 ± 5.7 | 0.953 ^a^ | 0.136 |
| Death within 90 days | 1 (0.7) | 1 (0.7) | 1.000 ^b^ | 0 |

Values represent mean ± standard deviation or number (%).

GDFT, goal-directed fluid therapy; RBC, red blood cell; FFP, fresh frozen plasma; Intraop, intraoperative; postop, postoperative; NA, not applicable; SMD, standardized mean difference.

†Mean ± standard deviation was obtained only for patients who received RBC transfusions.

p values were calculated using ^a^ student t-test; ^b^ chi-squared or Fisher’s exact test
