# Supplementary material for: Goal-directed fluid therapy on the postoperative complications of laparoscopic hepatobiliary or pancreatic surgery: An interventional comparative study
Source: PLoS One. 2024 Dec 18;19(12):e0315205. doi: 10.1371/journal.pone.0315205 (PMC11654985; doi:10.1371/journal.pone.0315205)
Supplement: S2 File — (DOCX) [file pone.0315205.s008.docx]

**연구계획서**

| **복강경으로 간담췌수술을 받는 환자에서 목표 설정 수액 요법이**  **수술 후 부작용 발생에 미치는 영향**  **The effect of goal-directed fluid therapy**  **on postoperative complications in patients**  **undergoing laparoscopic hepatobiliary or pancreas surgery** |
| --- |

**Version No. 2.3**

**분당서울대학교병원**

**마취통증의학과**

**오 아 영**

**연구 개요**

| 연구제목 | 복강경으로 간담췌 수술을 받는 환자에서 목표 설정 수액 요법이 수술 후 부작용 발생에 미치는 영향 |
| --- | --- |
|  | The effect of goal-directed fluid therapy on postoperative complications in patients undergoing laparoscopic hepatobiliary or pancreas surgery |
| 책임연구자 | 마취통증의학과 부교수 오아영 |
| 연구비 지원기관 | 에드워드 라이프사이언시스 코리아㈜ |

| 연구 목적 | □ 최근 중등도 이상의 고위험군 환자에서 수술 중 수액 요법에 Goal-directed fluid therapy (GDFT)를 적용하는 경우 기존의 방법에 비해 수술 후 합병증 발생의 감소, 중환자실 체류 기간 및 재원기간의 단축 등 환자의 유병률 감소에 기여한다는 것이 알려져 있다.  □ 복강경 수술은 최근 그 적용이 확대되고 있는 추세이지만 복강경 수술에 GDFT를 적용한 보고는 아직 없다.  □ 이에 본 연구에서는 복강경 간담췌 수술을 받는 환자를 대상으로 수술 중 GDFT protocol을 적용하고, 기존의 통상적인 방법으로 수액요법이 이루어진 환자들과 비교하여 수술 후 회복 및 합병증 발생에 차이가 있는지 알아보고자 한다. |
| --- | --- |
| 연구 설계 | 단일시험군과 과거대조군의 비교 연구 |
| 연구 기간 | IRB승인일 이후 2년 (1년 후 연차 지속 심의로 연장 예정) |
| 연구 대상 | 복강경으로 간담췌수술을 받는 환자 |
| 연구 대상자 수 | 실험군 환자 162명 (전향적 모집), 대조군 환자 228명 (후향적 조사) |
| 취약한 연구대상자 | 해당사항 없음. |
| 시험약 | 해당사항 없음. |
| 용법 및 용량 | 해당사항 없음. |
| 연구 방법 | □ 수술 중 수액 주입 방법에 따라 GDFT (GDFT군)와 기존의 방법(Control군)으로 수액 투여를 진행한 군으로 나누어 연구를 진행한다.  - GDFT군의 경우 EV1000 paltform 모니터를 통해 측정되는 침습적 동맥압 외에 cardiac index(CI), stroke volume index(SVI)와 stroke volume variation(SVV)를 토대로 GDFT protocol을 따라 수액 요법을 실시하고 아래 관찰항목을 조사한다.  - Control군의 경우 과거 복강경 간담췌수술을 받은 환자 중 Vigilio/FoTrac 모니터링을 사용하지 않고 수술이 진행된 환자를 선택하여 아래 관찰항목을 조사한다.  □ 주 관찰 항목  - Postoperative colplications  : pulmonary complication, wound complication, acute kidney injury, delirium, deep vein thrombosis, MI, stroke, sepsis, urinary tract infection, death  □ 부 관찰 항목  - 수술 중 투여된 총 수액 량  - 혈역학적 안정성  - 수술 후 재원 기간 및 수술 후 중환자실 재원 기간  - 수술 후 기관내튜브를 발관할 때 까지 걸린 시간  - 주술기 수혈 여부 및 수혈량 |
| 주요 선정기준 | [GDFT군] 2시간 이상 혹은 예상 실혈량 500 ml 이상의 복강경 간담췌수술이 예정된 20세 이상의 성인 남녀 환자  [Control군] 과거 복강경으로 간담췌수술을 받았던 20세 이상의 성인 남녀 환자 |
| 주요 선정  제외기준 | [GDFT군, control군 동일적용]  연구 참여에 동의하지 않은 환자  임산부, 수유부  복강경 수술 중 개복수술로 전환되는 환자  만성신질환으로 투석을 진행하고 있는 환자  Sepsis 환자  Pulmonary edema 환자  Congestive heart failure 환자  Severe coagulopathy가 동반된 환자  중증 전해질 이상 환자 (hypernatremia, hyperkalemia, hypermagnesemia, hyperchloremia)  부정맥을 지닌 환자 |
| 유효성 평가 | GDFT군에서 수술 중 적정량의 수액 투여가 이루어질 것이고, 이는 수술 후 합병증의 발생빈도로 감소로 나타날 것이다. |
| 안전성 평가 | 해당사항 없음 |
| 검사일정 | 해당사항 없음 |
| 통계적 분석방법 | 정규성 검정을 시행한 후 두 군간에 Student’s t-test 또는 Mann-Whitney U test, Chi-square test 분석을 시행한다. |
| 기대효과 및  예상결과 | 복강경 수술 시 수술 중 수액 요법에 대한 새로운 지침을 제시할 수 있고, 수술 후 부작용 발생 비율의 감소로 환자의 안전에 기여하고, 이로 인한 회복기간, 재원일수의 단축을 기대할 수 있어 환자의 만족도 향상과 환자의 삶의 질 개선에 기여할 수 있을 것으로 기대된다. |

**연구계획서**

1. **연구 제목**

복강경으로 간담췌 수술을 받는 환자에서 목표 설정 수액 요법이 수술 후 부작용 발생에 미치는 영향

1. **연구의 실시기관 명칭 및 주소**

분당서울대학교병원

경기도 성남시 분당구 구미로 178번길 82

1. **연구책임자 및 공동연구자 성명 및 직명**
2. **연구책임자**

마취통증의학과 오아영

1. **공동연구자**

마취통증의학과 나효석

마취통증의학과 구본옥

1. **연구 의뢰기관
   1) 연구 의뢰기관 명칭 및 주소**

해당사항 없음.
**2) 모니터요원 성명 및 직명**

해당사항 없음.

1. **연구비 지원기관**

에드워즈라이프사이언시스코리아㈜

서울특별시 강남구 봉은사로 112길 6

1. **예상연구기간**

IRB 승인 이후 2년 (1년 후 연차 지속 심의로 연장 예정)

1. **연구 대상 질환**

간담췌질환으로 복강경하에 수술을 받는 환자

1. **연구의 배경 및 목적**
2. **연구 배경**

수술 전후 수액 주입의 목적은 금식 및 수술과 관련된 조직 손상, 실혈 등에 따른 탈수를 피하고, 적절한 혈액 순환을 유지하여 조직으로의 혈류가 감소하지 않도록 유지하는 것이다. 수술 중 수액 공급의 부족은 물론 수액의 과도한 투여도 환자의 회복 및 예후에 중요한 영향을 끼치는 것은 잘 알려져 있다. 하지만 수술 중 필요한 적절한 수액의 양은 환자의 상태, 수술의 종류와 소요 시간 등에 따라 달라서 일괄적으로 정형화하기에는 어려움이 있다.

Goal-Directed Fluid Therapy (GDFT)는 직접적으로 심박출량 혹은 산소 전달에 관련된 지표들을 지속적으로 측정하여 투여될 수액의 양을 정하는 것으로 기존의 혈압, 심박수, 소변량 등에 의존하던 방법에 비해 더욱 적절한 수액공급을 가능하도록 해주는 것으로 알려졌다. GDFT를 위해 사용하는 방법은 여러 가지가 있을 수 있지만 그 중 EV1000 platform(Edwards Lifesciences, Irvine, CA, USA) 모니터를 사용하는 방법은 침습접 동맥압 감시를 하는 경우 추가의 침습적 시술 없이 연결하여 사용할 수 있다는 장점이 있다.

실제로 수술 중 GDFT를 사용한 경우 그렇지 않은 경우에 비해 수술 후 폐렴의 발생이나 상처 감염 등의 부작용이 줄었고, 중환자실 체류일 수 혹은 재원 일 수가 유의하게 줄었다는 보고가 있다 *{Critical Care 2015;19;261}*. 그 외 EV1000 platform이 아니더라도 수술 중 GDFT를 진행하는 경우 수술 후 30일 간 pneumonia, sepsis, intra-abdominal infection, catheter-related bloodstream infection, arrhythmia, heart failure, acute myocardial infarction, pulmonary embolism, ARDS, stroke, hepatic dysfunction을 포함하는 major postoperative complication이 약 20%정도 감소하였다 {*Critical Care 2010:14; R118}*. 부위마취 하에 hip replacement surgery를 진행하는 경우에도 abdominal infection, urinary infection, significant hypotension, 수혈이 필요한 anemia와 같은 complication 역시 GDFT군에서 덜 발생하였다 {Crit Care. 2011;15(3):R132}. 또한 postoperative lactate level이 GDFT에 의해 감소함을 알 수 있었다 {Anesth Analg. Oct 2010;111(4):910-914}.

복강경 수술은 기존의 방법에 비해 환자의 회복 및 예후에 유리한 것으로 알려져 있고 그 적응증이 점점 늘어나고 있는 추세이다. 하지만 복강경 수술 중의 적절한 수액 요법에 대해서는 많이 알려진 바가 없다. GDFT 에 관해서도 복강경 수술에 대해 적용한 경우는 아직 찾아 볼 수 없다. 이에 본 연구에서는 복강경으로 간담췌 수술을 받는 환자를 대상으로 GDFT를 적용하여 기존의 방법에 비해 환자의 회복에 어떤 영향을 미치는지를 알아보고자 한다.

1. **연구 가설 및 목적**

본 연구를 통하여 복강경 간담췌 수술 중 수액 요법을 실시하는 경우 GDFT가 기존의 방법에 비해 수술 중 더 안정적인 혈역학적 유지를 가능하게 하고, 수술 후 합병증의 발생 빈도를 감소시켜 환자의 수술 후 재원 기간을 단축시킬 수 있음을 보일 수 있다면 복강경 수술 중의 수액 요법에 관해 새로운 지침을 제시할 수 있을 것이다. 또한 수술 후 환자의 회복에 긍정적인 영향으로 환자의 안전 향상에 기여할 수 있을 것이다.

1. **임상시험용 의약품 코드명(또는 주성분의 일반명), 원료약품의 분량, 제형 등**

해당사항 없음.

1. **연구대상자의 선정 기준, 제외기준, 목표한 대상자 수 및 산출 근거**
2. **선정기준**

[GDFT군] 2시간 이상 혹은 예상 실혈량 500 ml 이상의 복강경 간담췌수술이 예정된 20세 이상의 성인 남녀 환자

[Control군] 과거 복강경으로 간담췌수술을 받았던 20세 이상의 성인 남녀 환자

1. **제외기준**

**[GDFT군, control군 동일적용]**

연구 참여에 동의하지 않은 환자

임산부, 수유부

복강경 수술 중 개복수술로 전환되는 환자

만성신질환으로 투석을 진행하고 있는 환자

Sepsis 환자

Pulmonary edema 환자

Congestive heart failure 환자

Severe coagulopathy가 동반된 환자

중증 전해질 이상 환자 (hypernatremia, hyperkalemia, hypermagnesemia, hyperchloremia)

부정맥을 지닌 환자

1. **목표한 대상자 수 및 산출 근거**

기존에 개복수술을 받는 환자를 대상으로 GDFT를 시행한 경우 수술 후 합병증의 빈도가 39.8%에서 24.8%로 약 15% 감소하였다는 연구 결과가 있다 *{Critical Care 2015;19;261}.*

분당서울대학교병원에서 2015년에 GDFT를 시행하지 않고 간담췌질환으로 복강경 수술을 받은 179명의 환자 중 본 연구에서 조사하려는 합병증의 발병률은 약 31%로 조사되었다.

동일 수술에서 GDFT를 시행하는 경우 본 병원에서 조사된 31%의 합병증 발병률을 17%까지 감소시킬 수 있다고 가정하는 경우, α=0.05, β=0.8, 각 군별로 145명 씩, 총 290명의 환자가 요구된다. 중도탈락률을 고려하면 총 324명의 환자가 필요하다.

GDFT를 시행할 예정인 GDFT군 162명은 전향적 모집이 될 것이고, 이에 대응하는 Control군 228명은 후향적 모집을 통해 진행할 예정이다.

1. **연구 대상자 모집 계획**

GDFT군의 경우 간담췌질환으로 복강경수술이 예정된 환자 중 사전에 동의를 한 환자를 대상으로 하는 환자군 연구이므로 별도의 공고를 통해 모집하지 않는다.

Control군의 경우 후향적 의무기록을 조사하는 연구이므로 별도의 공고가 필요 없다.

1. **연구 방법**
2. **구체적인 연구방법**

**[GDFT 군]**

- 수술 전 연구 대상이 되는 환자를 선정하여 연구에 대한 정보를 제공한 후 동의서를 취득한다. 동의서 취득이 완료된 환자는 모두 수술 중 GDFT를 사용하는 군(GDFT군)으로 배정된다.
- 수술 당일 환자가 수술장에 도착하면 수술장 입구의 전실에서 midazolam 0.03 mg/kg IVS 후 수술장에 입실한다. 수술장 입실 후 심전도, 비침습적 혈압계, 맥박산소포화도 감시 장치 등을 부착한 후 마취 유도를 시작한다. 마취 유도는 remifentanil, propofol, desflurane, rocuronium 으로 하고 마취 유지는 remifentanil, desflurane, rocuronium 으로 한다. 마취 심도의 측정을 위해 BIS sensor를 부착하고 BIS 수치 40-60을 유지할 수 있도록 마취 심도를 조절한다.
- 전신마취 중 ventilator는 tidal volume이 8ml/kg을 넘지 않는 범위에서 동맥혈가스 검사의 PaCO_2_ 가 35-40 mmHg 사이에서 유지되도록 respiratory rate와 함께 조절한다. Positive end-expiratory pressure는 마취과 의사의 판단에 따라 필요한 경우 적용한다.
- 침습적 동맥압 감시를 위해 동맥관을 거치하고 GDFT군에 배정된 환자의 경우 이를 FloTrac sensor를 이용하여 EV1000 platform(Edwards Lifesciences, Irvine, CA, USA) (Fig. 1)모니터에 연결하여 지속적 동맥압 외에 cardiac index(CI), stroke volume index(SVI)와 stroke volume variation(SVV)을 지속 감시한다. 빠른 수액 주입 혹은 약제의 투입을 위해 18 G 카테터로 추가의 정맥로를 확보한다. 추가로 식도 체온계와 Foley 카테터를 거치한 수 수술을 시작한다.
- 수술 중 수액의 주입은 다음과 같은 방법으로 한다.

기본 수액 주입 속도는 정질액 4-5 ml/kg/hr로 하고 수액 주입의 목표는 심박수 60-100/min, 수술 전 수축기 혈압의 ± 30%이내, SV reduction 10% 이하로 유지되도록 한다.

수술 전 수축기 혈압이 30% 이상 저하되는 경우 SV과 CI를 토대로 수액의 투여, vasoconstrictor의 투여, inotropics의 투여를 결정한다. 상세 protocol은 아래 Fig. 2.에 따른다. 수술 중 투여되는 교질액은 6% hydroxyethyl starch 130/0.4 in an isotonic electrolyte injection 제제를 사용하고 투여할 수 있는 최대량은 수술 중 1500ml가 넘지 않도록 한다.


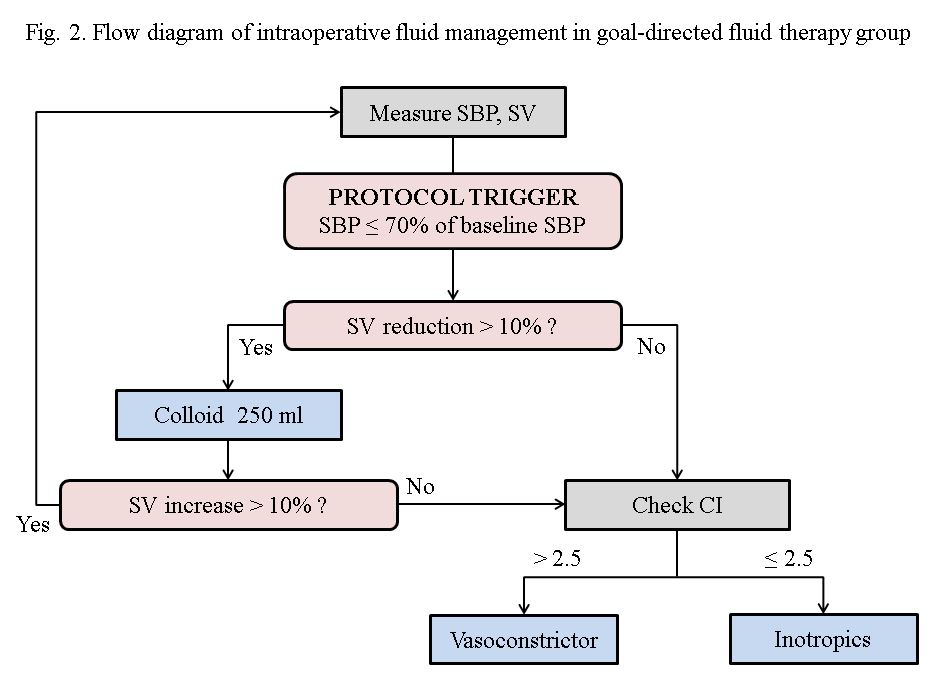


- 수술 중 수혈은 다음과 같이 시행한다.

RBC: 수술 중 출혈의 결과 Hb수치가 8 g/dl 이하로 저하된 경우 또는 지속적인 대량 출혈이 예상되는 경우. 단, coronary disease가 동반된 환자의 경우 심전도 이상, 혈압의 지속적인 저하, 적정 혈량에도 빈맥이 지속되는 경우 Hb수치가 8-10 g/dl인 경우 RBC 수혈을 진행할 수 있다.

FFP: 수술 중 출혈로 인해 RBC가 5 units 이상 투여된 경우 혈액응고검사에서 PT-INR이 2.0이상이거나 aPTT가 60 sec 이상 지연된 경우

Platelets: 수술 중 출혈로 인해 RBC가 10units 이상 투여되고 CBC 검사에서 platelet 수치가 50,000/mm^3^이하로 저하된 경우

**[Control 군]**

- 2012년 1월 1일부터 2015년 12월 31일 기간 동안 연구 대상이 되는 환자를 후향적으로 선정하여 Control군으로 배정한다.
- Control군으로 배정된 환자의 의무기록을 검토하여 해당 관찰항목을 조사한다.

1. **무작위 배정 및 눈가림 방법**

- 무작위 배정

GDFT군으로 배정되어 GDFT를 진행하는 환자의 경우 연구 승인 후 전향적으로 이루어지고, Control군으로 배정되는 환자는 조건에 일치하는 환자를 2012년 1월 1일부터 2015년 12월 31일까지 후향적으로 조사하게 되므로 무작위 배정은 해당사항이 없다. 연구에 동의한 환자는 모두 GDFT군으로 배정된다.

- 눈가림

해당사항이 없음.

1. **관찰항목 및 관찰검사방법**

• Primary outcome

Various postoperative complications

- Pulmonary complication

- Wound complication

- Acute kidney injury

- Delirium

- Deep vein thrombosis

- Myocardiac infarction

- Stroke

- Sepsis

- Urinary tract infection

- Death

: 수술 후 퇴원 기간까지 해당 합병증의 발병 유무를 기록한다.

: 수술 후 입원기간이 90일을 초과하는 경우 90일까지만 합병증 발생 유무를 조사한다.

• Secondary outcome

- 수술 중 투여된 총 수액 량: 정질액, 교질액

- 혈역학적 안정성: 저혈압, 심근수축제, 승압제의 투여 횟수 및 양

- 수술 후 재원 기간 및 수술 후 중환자실 재원 기간

- Extubation까지의 시간

- 주술기 수혈 여부 및 수혈량

- 수술 후 90일 이내 재입원 여부 및 사유

1. **효과 평가기준**

GDFT군에서 수술 중 적정량의 수액 투여가 이루어질 것이고, 이는 수술 후 합병증의 발생빈도 감소로 나타날 것이다.

1. **기존 연구와의 차별점**

기존 연구는 주로 중환자실의 환자나 개복술을 진행하는 환자에서의 수액 요법을 주로 취급하였다. 본 연구에서는 복강경으로 수술을 진행하는 환자를 대상으로 하므로 향후 복강경하에 수술을 받게되는 환자의 수액 요법에 새로운 지침을 제시할 수 있는 근거가 될 것이다.

1. **연구대상자의 이익과 위험**

연구대상자가 이번 연구에 참여하였을 때 직접적으로 얻을 수 있는 이익은 없다. 또한 제공되는 마취 및 수술 방법은 기존의 진료과정에서 변화되는 사항이 없다. 다만, 연구 개시 후 EV1000 platform을 이용한 모니터 하에 GDFT를 시행하면서 수술 후 합병증이 감소할 것으로 기대하고 있으므로 이에 따르는 간접적인 이익은 발생할 수 있다.

1. **중지∙탈락 기준**

- 동의가 철회된 환자
- 수술 방법이 복강경수술에서 개복술로 변경된 환자
- EV1000 platform 모니터가 유지되지 못하는 경우

1. **부작용을 포함한 안전성의 평가기준, 평가 방법 및 보고 방법**

- 해당사항 없음.

1. **자료안전성 모니터링 계획**

책임연구자가 해당 연구에 대해 지속적으로 모니터링을 실시한다.

임상 연구 중 발생한 예상치 못한 문제나 계획서 미 준수 등의 사례가 있는 경우 책임연구자는 이를 분당서울대학교 IRB에 보고한다.

- 모니터링 책임자: 오아영
- 모니터링 주기

모니터링 책임자는 최초 10명의 연구대상자가 등록된 후 자료안전선 모니터링을 실시하고 이후에는 매 30명의 연구대상자 등록 시 마다 자료안전성 모니터링을 실시한다.

- 모니터링 절차
  - 이상약물반응보고(fatal/life-threatening): 사망이나 생명을 위협하는 사례는 7일 이내(초기보고) + 8일이내(추적보고)
  - 이상약물반응보고(not fatal/life-threatening): 그 외의(입원 또는 입원기간의 연장, 지속적인 또는 중대한 불구나 기능저하를 초래, 선천성 기형 또는 이상을 초래, 중요한 의학적 사건, 기타) 사례는 15일 이내
  - 예상하지 못한 문제 보고: 15일 이내
  - 중대한 미준수 사례보고: 15일 이내
  - 사소한 미준수 사례보고: 정기보고주기별

1. **자료 분석 및 통계 분석 방법**

- 정규성 검사는 Kolmogorov-Smirnov test 시행

- 연속성 정규분포하는 자료는 Student’s t-test 와 Bonferroni correction 시행

- 연속성 비정규분포 자료는 Wilcoxon test 시행

- 이분분포하는 자료는 chi-squared test와 Fisher’s exact test 시행

- P 값이 0.05 이하일 때 유의한 결과로 판단.
- GDFT군과 control군의 basal characteristics에 차이가 발생하는 경우 두 군의 평형을 맞추기 위하여 propensity matching을 시행한 후 paired t-test, McNemar test를 연속성, 이분분포 자료에 맞추어서 시행하여 분석.

1. **연구수행일정표**

- IRB 승인일 ~ 18 개월: data 수집
- 19~22 개월: data 분석 및 필요 시 추가 data 수집
- 23~24 개월: 논문 작성 및 제출

1. **연구대상자의 안전보호를 위한 대책**
2. **연구의 윤리성 확보를 위한 기본 방안**

본 임상 연구는 분당서울대학교병원의 생명윤리심의위원회의 심의를 거쳐 실시되며 헬싱키 선언과 생명 윤리 및 안전에 관한 법률을 준수한다. 또한 IRB 승인 후 본 연구는 진행될 예정이다.

본 연구에 참여하는 대상자의 정보는 오직 임상 연구에 참여하는 의료진에 의해 연구의 목적으로만 사용되도록 한다. 단, 연구의 절차 및 자료의 확인을 위하여 본 병원의 임상시험센터 및 연구대상자 보호센터에서는 필요 시 해당 연구대상자의 기록을 접할 수 있으나 이는 법적으로 허용하는 한도 내에서 행해질 것이다. 향후 임상시험의 결과가 출판되는 경우에도 해당 연구 대상자의 신원은 절대 노출되지 않을 것이다.

1. **연구대상자의 동의 과정**

- 연구대상자에게 설명하고 동의를 취득할 연구자: 책임, 담당, 또는 공동 연구자

- 동의를 제공할 자: 연구대상자

- 연구 설명 과정과 동의 취득 과정 사이의 대기 시간: 최소 하루 전 까지 제공하고 동의서를 확보

- 강제 또는 부당한 영향의 가능성을 최소화시킬 방법: 설명서 제공 후 충분한 숙지의 시간을 갖게 하고, 언제든지 자의로 중단할 수 있으며, 임상연구의 불참이 진료에 불이익을 주지 않는 다는 것을 설명서에 기술.

- 연구 설명 과정과 동의 취득 과정에서 연구자가 사용하는 언어: 한국어

- 연구대상자 또는 대리인이 이해할 수 있는 언어: 한국어를 모국어로 사용하는 자만 대상으로 한다.

- 연구대상자 또는 대리인에게 제공되는 정보와 동의서 서식: 별첨

1. **연구대상자의 보상 방안**

별첨.

1. **연구대상자의 개인정보보호 방안**

환자의 의무기록번호 및 병리 번호는 책임 연구자의 책임하에 별도의 파일로 보관하며 이를 코드화하여 연구데이터를 통하여 개인 신상 확인이 불가능하도록 관리한다. 또는 연구데이터는 패스워드가 걸린 파일에 저장하여 잠금 장치가 있는 연구실에 보관하도록 한다.

생명윤리법 시행규칙 제15조에 따라 연구 관련 기록을 연구가 종료된 시점부터 3년간 보관하여야 하며, 보관기관이 지난 문서는 개인정보보호법 시행령 제16조에 따라 파기할 것이다.

1. **취약한 연구대상자를 포함하는 경우 추가적인 보호조치 방안**

해당사항 없음.

1. **인체유래물의 보관 및 폐기 방법**

해당사항 없음.

1. **참고 문헌**

- Benes J, Chytra I, Altmann P, Hluchy M, Kasal E, et al. Intraoperative fluid optimization using stroke volume variation in high risk surgical patients: results of prospective randomized study. Crit Care 2010; 14: R118.
- Benes J, Giglio M, Brienza N, Michard F. The effect of goal-directed fluid therapy based dynamic parameters on post-surgical outcome: a meta-analysis of randomized controlled trials. Crit Care 2014; 18: 584.
- Donati A, Loggi S, Preiser JC, et al. Goal-directed intraoperative therapy reduces morbidity and length of hospital stay in high-risk surgical patients. Chest 2007; 132(6): 1817-24.
- Gan TJ, Soppitt A, Maroof M, et al. Goal-directed intraoperative fluid administration reduces length of hospital stay after major surgery. Anesthesiol 2002; 97(4): 820-6.
- Mayer M, Boldt J, Mengistu AM, Rohm KD, Suttner S. Goal-directed intraoperative therapy based on autocalibrated arterial pressure waveform analysis reduces hospital stay in high-risk surgical patients: a randomized, controlled trial. Crit Care 2010; 14: R18.
- Nisanevich V, Felsenstein I, Almogy G, et al. Effect of intraopertaive fluid management on outcome after intraabdominal surgery. Anesthesiol 2005; 103: 25-32.
- Noblett SE, Snowden CP, Shenton BK, HOrgan AF. Randomized clinical trial assessing the effect of Doppler-optimized fluid management on outcome after elective colorectal resection. Br J Surg 2006; 93(9): 1069-76.
- Yates DRA, Davies SJ, Milner HE, Wilson RJT. Crystalloid or colloid for goal-directed fluid therapy in colorectal surgery. Br J Anaesth 2014; 111(2): 281-9.
- Cecconi M, Fasano N, Langiano N, et al. Goal-directed haemodynamic therapy during elective total hip arthroplasty under regional anaesthesia. Crit Care 2011; 15: R132
- Forget P, Lois F, de Kock M. Goal-directed fluid management based on the pulse oximeter-derived pleth variability index reduces lactate levels and improves fluid management. Anesth Analg 2010; 111: 910-4
- Cannesson M, Ramsingh D, Rinehart J, et al. Perioperative goal-directed therapy and postoperative outcomes in patients undergoing high-risk abdominal surgery: a historical-prospective, comparative effectiveness study. Crit Care 2015; 19: 261
- Liu F, Zhu S, Ji Q, Li W, Liu J. The impact of intra-abdominal pressure on the stroke volume variation and plethysmographic variability index in patients undergoing laparoscopic cholecystectomy. Bioscience trends 2015; 9: 129-33.
- Munoz JL, Gabaldon T, Miranda E, et al. Goal-Directed Fluid Therapy on Laparoscopic Sleeve Gastrectomy in Morbidly Obese Patients. Obesity surgery 2016.
- Kuper M, Gold SJ, Callow C, et al. Intraoperative fluid management guided by oesophageal Doppler monitoring. Bmj 2011; 342: d3016.
