# Supplementary material for: Goal-directed fluid therapy on the postoperative complications of laparoscopic hepatobiliary or pancreatic surgery: An interventional comparative study
Source: PLoS One. 2024 Dec 18;19(12):e0315205. doi: 10.1371/journal.pone.0315205 (PMC11654985; doi:10.1371/journal.pone.0315205)
Supplement: S3 File — (DOCX) [file pone.0315205.s009.docx]

**Research Protocol**

1. **Research Title**

The Impact of Goal-Directed Fluid Therapy on Postoperative Complications in Patients Undergoing Laparoscopic Hepatobiliary or Pancreatic Surgery

1. **Name and Address of the Research Institution**

Seoul National University Bundang Hospital

82 Gumi-ro 178beon-gil, Bundang-gu, Seongnam, Gyeonggi-do, South Korea

1. **Principal Investigator and Co-Investigators**
2. **Principal Investigator**

Ah-Young Oh, Department of Anesthesiology and Pain Medicine

1. **Co-Investigators**

Hyo-Seok Na, Department of Anesthesiology and Pain Medicine

Bon-Wook Koo, Department of Anesthesiology and Pain Medicine

1. **Research Sponsor**
2. Name and Address of Research Sponsor

Not Applicable

1. Name and Title of Monitors

Not Applicable

1. **Funding Agency**

Edwards Lifesciences Korea, Ltd.

6 Gil 112-ro, Bongeunsa-ro, Gangnam-gu, Seoul, South Korea

1. **Expected Research Duration**

Two years following IRB approval (with the possibility of extension after the first year's annual review).

1. **Target Disease for Research**

Patients undergoing laparoscopic hepatobiliary or pancreatic surgery for hepatobiliary or pancreatic diseases.

1. **Research Background and Objectives**
2. **Research Background**

The purpose of fluid administration before and after surgery is to prevent dehydration due to fasting and surgical tissue damage, bleeding, and to maintain adequate blood circulation to prevent decreased tissue perfusion. It is well known that both insufficient and excessive fluid supply during surgery can have a significant impact on patient recovery and outcomes. However, determining the appropriate amount of fluid during surgery can be challenging, as it depends on factors such as the patient's condition, the type and duration of the surgery.

Goal-Directed Fluid Therapy (GDFT) is a method of determining fluid administration based on continuously measuring indicators directly related to cardiac output or oxygen delivery, in contrast to the traditional reliance on blood pressure, heart rate, and urine output. Using the EV1000 platform (Edwards Lifesciences, Irvine, CA, USA) is one method to apply GDFT, and it has the advantage of enabling continuous monitoring without additional invasive procedures. Studies have shown that GDFT reduces postoperative complications, such as pneumonia, sepsis, and infections, and decreases ICU stay or hospitalization days significantly.

While the benefits of laparoscopic surgery in terms of patient recovery and outcomes are well-established, there is limited information on appropriate fluid management during laparoscopic procedures. There are no documented applications of GDFT in laparoscopic surgery. Therefore, this study aims to investigate the impact of GDFT on patient recovery in laparoscopic hepatobiliary or pancreatic surgery compared to conventional fluid management.

1. **Research Hypothesis and Objectives**

Through this study, we aim to demonstrate that GDFT, when applied to laparoscopic hepatobiliary or pancreatic surgery, enables more stable hemodynamic maintenance during surgery than the conventional method. Furthermore, GDFT may reduce the frequency of postoperative complications and, consequently, shorten the postoperative hospitalization period, leading to improved patient safety. If these outcomes are achieved, this research may provide new guidelines for fluid management in laparoscopic surgery and positively influence patient recovery.

1. **The document states**

Not applicable

1. **Selection Criteria, Exclusion Criteria, Targeted Number of Participants, and Rationale**
2. **Inclusion criteria**

Patients scheduled for laparoscopic hepatobiliary or pancreatic surgery are selected for the study, and information about the research is provided, followed by obtaining informed consent. Patients who have completed the informed consent process are all assigned to the GDFT group.

1. Exclusion criteria

Exclusion Criteria

[Applies to both the GDFT group and the control group]

Patients who do not consent to participate in the study

Pregnant or lactating patients

Patients undergoing conversion from laparoscopic to open surgery during abdominal surgery

Patients with chronic kidney disease requiring dialysis

Patients with sepsis

Patients with pulmonary edema

Patients with congestive heart failure

Patients with severe coagulopathy

Patients with severe electrolyte imbalances (hypernatremia, hyperkalemia, hypermagnesemia, hyperchloremia)

Patients with arrhythmia

1. **Target Number of Participants and Rationale**

There is a study that shows a 15% reduction in the frequency of postoperative complications from 39.8% to 24.8% when GDFT was implemented in patients undergoing open surgery {Critical Care 2015;19;261}.

Among the 179 patients who underwent laparoscopic surgery for liver and pancreas disease without GDFT at Bundang Seoul National University Hospital in 2015, the incidence rate of complications to be investigated in this study was approximately 31%.

Assuming a reduction from 31% to 17% in the incidence rate of complications when GDFT is implemented in the same surgery, with α=0.05, β=0.8, and 145 patients per group, a total of 290 patients are required. Considering the dropout rate, a total of 324 patients are needed.

The GDFT group, consisting of 162 patients who will undergo GDFT, will be recruited prospectively, while the corresponding Control group of 228 patients will be recruited retrospectively.

1. **Participant Recruitment Plan**

For the GDFT group, patients scheduled for laparoscopic liver and pancreas surgery are included in the study without separate recruitment as they provide consent in advance.

For the Control group, retrospective medical record review is conducted; hence, no separate recruitment is required.

1. **Specific Research Methods**
2. **Specific study protocol**

On the day of surgery, when the patient arrives at the operating room, they receive midazolam at a dose of 0.03 mg/kg intravenously in the preoperative room just outside the operating room. After entering the operating room, the patient is equipped with an electrocardiogram, non-invasive blood pressure monitor, pulse oximetry, and other monitoring devices, and anesthesia induction begins. Anesthesia induction is performed using remifentanil, propofol, desflurane, and rocuronium, while anesthesia maintenance consists of remifentanil, desflurane, and rocuronium. To monitor the depth of anesthesia, a BIS sensor is attached, and anesthesia depth is adjusted to maintain BIS values between 40 and 60.

During general anesthesia, the ventilator is adjusted to maintain a tidal volume that does not exceed 8 ml/kg and keeps the PaCO2 within the range of 35-40 mmHg according to the arterial blood gas analysis. Positive end-expiratory pressure (PEEP) is applied if deemed necessary by the anesthetist and surgeon.

For invasive arterial pressure monitoring, an arterial catheter is inserted, and patients assigned to the GDFT group are connected to the FloTrac sensor using the EV1000 platform (Edwards Lifesciences, Irvine, CA, USA) for continuous arterial pressure monitoring. In addition to continuous arterial pressure, the system continuously monitors cardiac index (CI), stroke volume index (SVI), and stroke volume variation (SVV). An additional intravenous line with an 18G catheter is secured for rapid fluid administration or drug infusion. Esophageal temperature monitoring and a Foley catheter are also placed before the surgery begins.

The fluid administration during the surgery is performed as follows: The basal fluid infusion rate is set at 4-5 ml/kg/hr of crystalloid, and the fluid administration target is to maintain a heart rate of 60-100/min, systolic blood pressure within ±30% of preoperative values, and a stroke volume reduction of less than 10%.

In cases where the systolic blood pressure decreases by more than 30% from the preoperative value, fluid administration, vasoconstrictor use, or inotropic agent use is determined based on SV and CI. Detailed protocols are followed as illustrated in Figure 2. Colloid solutions used for intraoperative fluid administration consist of 6% hydroxyethyl starch 130/0.4 in an isotonic electrolyte solution, and the maximum allowable volume administered does not exceed 1500 ml during the surgery.


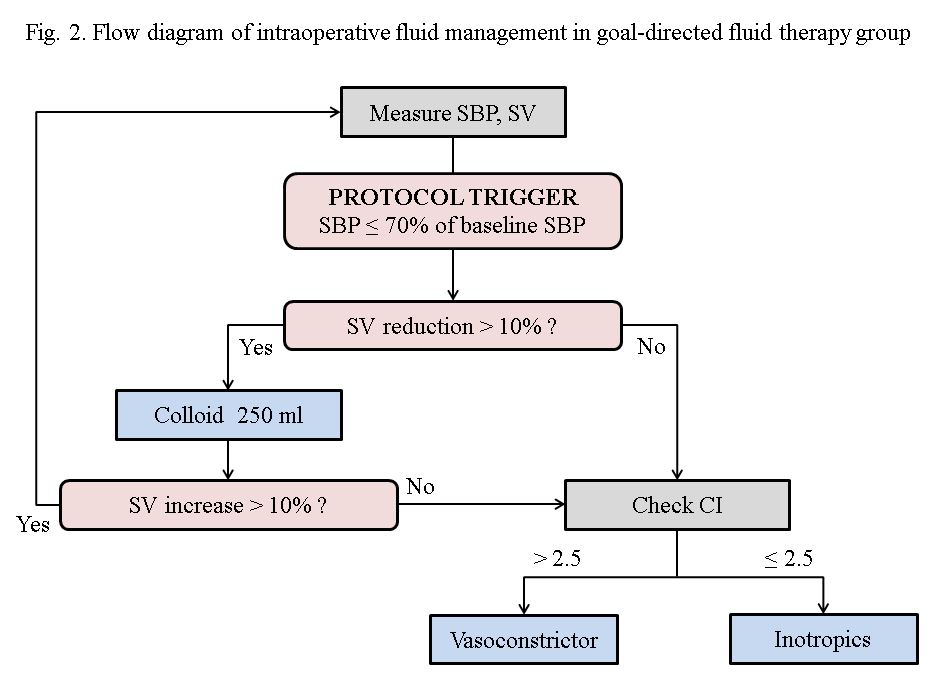


Blood transfusions during surgery

Red Blood Cells (RBC): Administered when the hemoglobin (Hb) level drops to 8 g/dl or below due to intraoperative bleeding or when continuous significant bleeding is expected. In cases where patients with coronary artery disease exhibit abnormal ECG, sustained low blood pressure, and persistent bradycardia despite adequate blood volume, RBC transfusion may be initiated with Hb levels between 8-10 g/dl.

Fresh Frozen Plasma (FFP): Administered when five or more units of RBC are given due to intraoperative bleeding, or when the blood coagulation test reveals a PT-INR of 2.0 or higher or an aPTT of 60 seconds or longer.

Platelets: Administered when ten or more units of RBC are given due to intraoperative bleeding, and a CBC test shows a platelet count of 50,000/mm3 or less.

**[Control Group]**

Patients included in the study from January 1, 2012, to December 31, 2015, are retrospectively selected for the Control Group.

The medical records of patients in the Control Group are reviewed to investigate the relevant observational data.

**2) Randomization and Blinding Method**

□ Randomization

For patients in the GDFT Group who undergo GDFT during surgery, it is conducted prospectively following research approval. Patients assigned to the Control Group are identified by retrospective data matching based on the conditions mentioned, covering the period from January 1, 2012, to December 31, 2015. Randomization does not apply since all patients who have consented to participate are assigned to the GDFT Group.

□ Blinding

Not applicable.

1. **Observational Variables and Observation Methods**

Primary Outcome: Various postoperative complications are recorded up to the date of discharge following surgery.

If the postoperative hospitalization exceeds 90 days, complications are investigated only up to 90 days.

Secondary Outcome:

Total volume of fluids administered during surgery: crystalloid, colloid

Hemodynamic stability: the number and amount of vasopressors, inotropes, and positive inotropic agents administered

Postoperative length of stay and ICU admission

Time to extubation

Blood transfusion during surgery and transfusion volume

Readmission within 90 days of surgery and reasons for readmission

1. **Evaluation Criteria for Effectiveness**

It is expected that adequate fluid administration during surgery in the GDFT Group will be associated with a decrease in the frequency of postoperative complications.

1. **Differentiating Factors from Previous Research**

Previous research has mainly focused on fluid therapy in patients admitted to the ICU or undergoing open surgery. However, this study targets patients undergoing laparoscopic surgery in the abdominal cavity, offering the potential to provide new guidelines for fluid therapy for future patients receiving laparoscopic surgery.

1. **Benefits and Risks to Research Subjects**

Participants in this study will not receive direct benefits. The provided anesthetic and surgical methods do not introduce any changes from the standard clinical procedures. Nevertheless, with the implementation of GDFT under the monitoring of the EV1000 platform, a reduction in postoperative complications is anticipated, leading to potential indirect benefits.

1. **Criteria for Discontinuation or Withdrawal**

Patients who withdraw their consent

Patients whose surgical approach changes from laparoscopic surgery to open surgery

When the EV1000 platform monitor cannot be maintained

1. **Criteria for Safety Evaluation, Assessment Methods, and Reporting**

Not applicable.

1. **Data Safety Monitoring Plan**

The Principal Investigator (PI) will continuously monitor the study. In cases of unforeseen problems, deviations from the protocol, etc., the PI will report these to the Institutional Review Board of Bundang Seoul National University Hospital.

Monitor: Dr. Ah-Young Oh

Monitoring Frequency:

Initial Data Safety Monitoring after the enrollment of the first 10 research subjects.

Subsequent Data Safety Monitoring conducted with every 30 research subjects enrolled.

Monitoring Procedures:

Report of Fatal/Life-Threatening Adverse Reactions: Within 7 days (initial report) and an additional 8 days (follow-up report).

Report of Non-Fatal/Life-Threatening Adverse Reactions (all other significant adverse events, including admission, prolonged admission, persistence, functional disability, congenital anomaly or birth defect, important medical events, other): Within 15 days.

Unexpected Problems Involving Risks to Subjects or Others: Within 15 days.

Reporting of Significant Protocol Deviations: Per the regular reporting schedule.

1. **Data Analysis and Statistical Methods**

Normality testing using the Kolmogorov-Smirnov test.

For continuous variables following a normal distribution, Student's t-test and Bonferroni correction are applied.

For continuous variables not following a normal distribution, the Wilcoxon test is used.

For dichotomous variables, the chi-squared test and Fisher's exact test are performed.

A p-value less than or equal to 0.05 is considered statistically significant.

If there are differences in baseline characteristics between the GDFT and Control groups, propensity matching is conducted to balance the two groups. Paired t-tests and McNemar tests are then used for analysis based on the nature of the variables.

1. **Study Timeline**

IRB Approval to 18 months: Data collection

19-22 months: Data analysis and additional data collection as needed

23-24 months: Paper writing and submission

1. **Measures to Ensure the Safety of Research Subjects**
2. **Basic Ethical Safeguards for Research**

This clinical study is conducted after obtaining approval from the Institutional Review Board (IRB) of Bundang Seoul National University Hospital and in compliance with the Helsinki Declaration and the Law on Life Ethics and Safety. The study will proceed following IRB approval. Information of research subjects participating in this study will be used solely for the purpose of research by the medical professionals involved in the clinical study. However, for procedural verification and data confirmation, the Clinical Trials Center and Research Subject Protection Center of our hospital may access the records of relevant research subjects if necessary, within the legal limits. Furthermore, when the results of the clinical trial are published, the identity of the research subjects will never be disclosed.

1. **Informed Consent Process**

Person responsible for explaining the research and obtaining consent: Principal, co-investigator, or assigned investigator

Person providing consent: Research subjects

Waiting period between the explanation and obtaining consent: Consent is obtained at least one day after providing explanations and receiving the informed consent form.

Methods to minimize the possibility of coercion or undue influence: Research subjects are provided with a thorough explanation, are allowed to withdraw their consent at any time, and it is explained in the information sheet that non-participation in the clinical study will not result in any disadvantages in their medical treatment.

Language used by the investigator in the explanation process and consent acquisition: Korean

Language that research subjects or their legal representatives can understand: Only individuals for whom Korean is their native language are included.

Information and consent form provided to research subjects: Attached (Not included in the document).

1. **Protection of Personal Information of Research Subjects**

The patient's medical record number and pathology number are stored separately in a file under the supervision of the Principal Investigator. The information is coded to ensure that personal identification through research data is impossible. Alternatively, research data is stored in password-protected files and kept in a secured laboratory with locking devices. According to Article 15 of the Enforcement Regulations of the Life Ethics Act, research-related records should be retained for three years from the end of the study, and expired documents will be destroyed in accordance with Article 16 of the Enforcement Decree of the Personal Information Protection Act.

1. **Additional Protective Measures When Including Vulnerable Research Subjects**

Not applicable.

1. **Storage and Disposal Methods for Human-Derived Materials**

Not applicable

1. References

Benes J, Chytra I, Altmann P, Hluchy M, Kasal E, et al. Intraoperative fluid optimization using stroke volume variation in high risk surgical patients: results of prospective randomized study. Crit Care 2010; 14: R118.

Benes J, Giglio M, Brienza N, Michard F. The effect of goal-directed fluid therapy based dynamic parameters on post-surgical outcome: a meta-analysis of randomized controlled trials. Crit Care 2014; 18: 584.

Donati A, Loggi S, Preiser JC, et al. Goal-directed intraoperative therapy reduces morbidity and length of hospital stay in high-risk surgical patients. Chest 2007; 132(6): 1817-24.

Gan TJ, Soppitt A, Maroof M, et al. Goal-directed intraoperative fluid administration reduces length of hospital stay after major surgery. Anesthesiol 2002; 97(4): 820-6.

Mayer M, Boldt J, Mengistu AM, Rohm KD, Suttner S. Goal-directed intraoperative therapy based on autocalibrated arterial pressure waveform analysis reduces hospital stay in high-risk surgical patients: a randomized, controlled trial. Crit Care 2010; 14: R18.

Nisanevich V, Felsenstein I, Almogy G, et al. Effect of intraopertaive fluid management on outcome after intraabdominal surgery. Anesthesiol 2005; 103: 25-32.

Noblett SE, Snowden CP, Shenton BK, HOrgan AF. Randomized clinical trial assessing the effect of Doppler-optimized fluid management on outcome after elective colorectal resection. Br J Surg 2006; 93(9): 1069-76.

Yates DRA, Davies SJ, Milner HE, Wilson RJT. Crystalloid or colloid for goal-directed fluid therapy in colorectal surgery. Br J Anaesth 2014; 111(2): 281-9.

Cecconi M, Fasano N, Langiano N, et al. Goal-directed haemodynamic therapy during elective total hip arthroplasty under regional anaesthesia. Crit Care 2011; 15: R132

Forget P, Lois F, de Kock M. Goal-directed fluid management based on the pulse oximeter-derived pleth variability index reduces lactate levels and improves fluid management. Anesth Analg 2010; 111: 910-4

Cannesson M, Ramsingh D, Rinehart J, et al. Perioperative goal-directed therapy and postoperative outcomes in patients undergoing high-risk abdominal surgery: a historical-prospective, comparative effectiveness study. Crit Care 2015; 19: 261

Liu F, Zhu S, Ji Q, Li W, Liu J. The impact of intra-abdominal pressure on the stroke volume variation and plethysmographic variability index in patients undergoing laparoscopic cholecystectomy. Bioscience trends 2015; 9: 129-33.

Munoz JL, Gabaldon T, Miranda E, et al. Goal-Directed Fluid Therapy on Laparoscopic Sleeve Gastrectomy in Morbidly Obese Patients. Obesity surgery 2016.

Kuper M, Gold SJ, Callow C, et al. Intraoperative fluid management guided by oesophageal Doppler monitoring. Bmj 2011; 342: d3016.
